# Relationships between feeding behaviors and emotions: an electroencephalogram (EEG) frequency analysis study

**DOI:** 10.1186/s12576-022-00858-w

**Published:** 2023-03-03

**Authors:** Shintaro Kusunoki, Takako Fukuda, Saori Maeda, Chenjuan Yao, Takahiro Hasegawa, Tetsuya Akamatsu, Hiroshi Yoshimura

**Affiliations:** 1 Field of Food Science & Technology, Graduate School of Technology, Industrial & Social Sciences, Tokushima University Graduate School, 2-1, Minami-josanjima-cho, Tokushima, 770-8513 Japan; 2 Department of Molecular Oral Physiology, Institute of Biomedical Sciences, Tokushima University Graduate School, 3-18-15 Kuramoto, Tokushima, 770-8504 Japan

**Keywords:** Electroencephalogram, Theta frequency, Low-beta frequency, Feeding behavior, Emotion

## Abstract

Feeding behaviors may be easily affected by emotions, both being based on brain activity; however, the relationships between them have not been explicitly defined. In this study, we investigated how emotional environments modulate subjective feelings, brain activity, and feeding behaviors. Electroencephalogram (EEG) recordings were obtained from healthy participants in conditions of virtual comfortable space (CS) and uncomfortable space (UCS) while eating chocolate, and the times required for eating it were measured. We found that the more participants tended to feel comfortable under the CS, the more it took time to eat in the UCS. However, the EEG emergence patterns in the two virtual spaces varied across the individuals. Upon focusing on the theta and low-beta bands, the strength of the mental condition and eating times were found to be guided by these frequency bands. The results determined that the theta and low-beta bands are likely important and relevant waves for feeding behaviors under emotional circumstances, following alterations in mental conditions.

## Background

Proper nutrition plays a crucial role in maintaining healthy conditions, which gets established through good feeding circumstances. An adequate appetite is essential for an individual to have adequate feeding behaviors. In uncomfortable environments, such as noisy or musty odor conditions, we are likely to lose our appetite, whereas comfortable environments, such as with relaxing music or the pleasant smell of a dish, likely result in better appetite [1–3]. Thus, feeding behavior may be modulated by the environments during eating [4].

Emotions, such as comfort or discomfort, are mainly produced by neural activities in the limbic systems [5–7], and the limbic system activities modulate appetitive behaviors [8–10]. The feeding center in the hypothalamus is also a key area that controls appetite and eating behaviors, which is modulated by activities of the limbic system [9, 11–13]. Therefore, emotions seem to be especially crucial for good nutrition and feeding behaviors. Yet, the relationships between emotions, brain activities, and patterns of eating behavior have not been ascertained.

Electroencephalogram (EEG) signals recorded from the surface of the head are based on comprehensive electrical fields produced by individual neural activities in the brain. Therefore, specifically interpreting the mean of the EEG signals is difficult. Despite this limitation, EEG recording is an effective measure for the interpretation of mental conditions [14–18].

In the present study, we prepared comfortable and uncomfortable virtual spaces, and the EEG activities of human participants were recorded while eating in these spaces. The conditions in this study allowed the investigation of each circumstance’s effects on feeding behaviors by altering brain activities and emotions.

## Materials and methods

All the procedures performed in this study involving human participants were in accordance with the ethical standards of the institutional and/or national research committee and with the 1964 Declaration of Helsinki and its later amendments or comparable ethical standards. The institutional ethics committee of Tokushima University approved this study (No. 2575-2), and informed consent was obtained from all the participants.

### Participants and measuring instruments

**Fig. 1:**
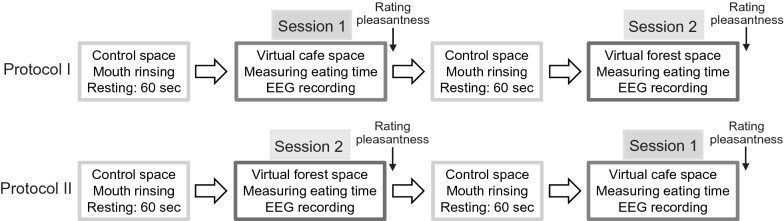
Flow chart of experiments. EEG recordings are performed during eating chocolate under the “virtual cafe space” (“Session 1”) or “virtual forest space” (“Session 2”), and the eating times were measured in subsequent sessions. Participants performed both “Protocol 1” and “Protocol 2.” Note that participants rested for 60 s before the first session, and between the first and second sessions. The differences between “Protocol I” and “Protocol II” are the orders of performing “Session 1” and “Session 2.”

Thirteen healthy participants (7 male and 6 female) with a mean (± standard deviation) age of 27.4 ± 9.5 years were included in the present experiments. We used milk chocolate (Morinaga & Co., Tokyo, Japan) as the food for the experiment, and all participants preferred chocolate. EEGs were recorded from the frontal region of the scalp (Fp1 and Fp2) using an Alphatec IV EEG recorder [Brain Function Research & Development Center (BFRDC), Osaka, Japan]. The sampling rate was 1024 Hz and the EEG signals were filtered between 1 Hz and 23 Hz. The common mode rejection ratio (CMRR) was greater than 60 dB. EEG epochs with artifacts were automatically identified, and artifact-free EEG epochs were used for the frequency analysis. Frequency analyses of the EEGs were performed using MinD Sensor V (BFRDC). During one session (“Session 1” and “Session 2”; see Fig. 1), the data were collected every 1 s. For convenience, frequency analysis at 1–3 Hz was excluded to avoid artifacts in the readings.

### Study procedure

Participants were seated in a body sonic chair (RFRESH1 Excellent, Tokyo, Japan) and listened to sounds. In front of the chair, a 150 cm × 100 cm screen was placed at a distance of 2 m for the participants to view the images. The images were projected onto the screen through a projector, EMP-1705 (EPSON, Tokyo, Japan), connected to a PC. Virtual scenes were formed using images and sounds. Scenes of “chocolate sweets in the cafe room” were prepared using photos of chocolate in a cafe room with consonant music. We named these scenes the “virtual cafe space.” Furthermore, scenes of dense thickets in a forest with bugs were prepared using photos of forests with many bugs and insect sounds. Those scenes were named the “virtual forest space.” A table was set on the body sonic chair, and the participants ate one piece of chocolate (3.5 g) freely upon sucking (but not chewing) while seated and viewing each of the virtual scenes, in succession.

The total eating time, from the start of eating to the end of swallowing, was measured using a stopwatch during each session. The details of the sessions are described below. After the session, the participant was asked to rate their feelings for pleasantness (with -5 being very unpleasant and + 5 being very pleasant). We termed these rates as the “score of pleasantness” in this study.

A flowchart of the experiment is shown in Fig. 1. In “Session 1,” the EEG was recorded during the “virtual cafe space,” while sucking on the chocolate and the eating time was measured. For “Session 2,” the EEG was recorded during the “virtual forest space,” while sucking on chocolate and the eating time was measured. Before every session, the participants rested for 60 s under a control condition with no image or sound. During this resting time, the participants rinsed their mouths with water. Two kinds of protocols were implemented. “Protocol I” was composed of: Resting, Session 1, Resting, and Session 2, in the stated order. “Protocol II” was composed in the following particular order: Resting, Session 2, Resting, and Session 1. The participants performed each protocol 1–2 h after the meal on separate days.

### Analyses of EEGs

The EEGs were recorded from the start of eating to the end of swallowing, and the data were collected every 1 s. Individual EEG frequencies were analyzed from 4 to 23 Hz per second. The “occupancy rate” for each frequency was calculated as follows: The power of each frequency was divided by the summed power of all frequencies, and the ratio was defined as the unit occupancy rate (%). Activity at each frequency was first assessed using the unit occupancy rate per second. The “unit occupancy rates” through one session were then averaged, and we adopted those data for the “occupancy rate” (%) in this study. Details of the EEG recordings and data analysis have been described in our previous report [19].

## Results

In a few sessions, electromyograms (EMGs) were recorded from the surface of the skin and around the area of the masseter muscles and suprahyoid muscles to identify the timing of when eating started and when the swallowing ended. The identification of these timings enabled us to measure the eating times precisely. When comparing “Protocol I” with “Protocol II,” the difference was in the order of performing “Session 1” and “Session 2.” For all participants, the experiments were executed according to both “Protocol 1” and “Protocol 2” (Fig. 1). The purpose of changing the order of the sessions was to check if the orders affected the measurement results acquired from the participants. As expected, changing the order of performing “Session 1” and “Session 2” did not affect the relative differences in pleasantness scores along with the relative differences in eating times between the two virtual circumstances. For this reason, the “pleasantness score” and “eating times” obtained both in “Session 1” and “Session 2” were used for analyses, regardless of the order of the sessions. Thus, values obtained from the two sessions were averaged, respectively, for every participant and adopted as the value of each participant. Accordingly, EEG data were analyzed regardless of the order of the session.

### Emotions and feeding behaviors

**Fig. 2:**
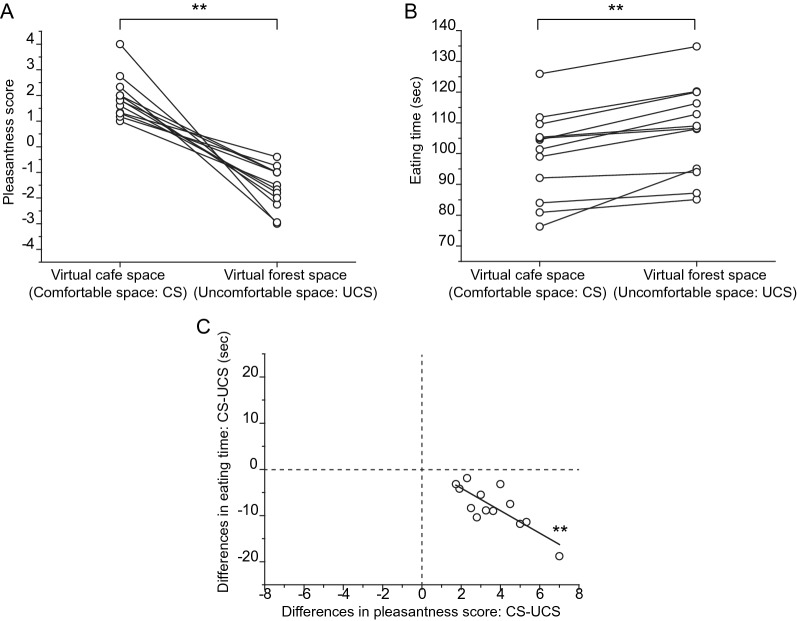
Relationships between pleasantness feelings and feeding behaviors under emotional circumstances. **A** Comparison of pleasantness scores (scores of − 5 −  + 5, where − 5 = extreme unpleasantness, 0 = no feeling of pleasantness, and + 5 = extreme pleasantness) under “Virtual cafe space” (= comfortable space: CS) and under the “Virtual forest space” (= uncomfortable space: UCS). Individual scores of the subjective feelings of pleasantness under the two spaces while eating chocolate were plotted. A set of two points are connected by a line. **B** Comparison of eating times under the CS and the UCS. Individual lengths of time for eating chocolate from the start of eating to the end of swallowing under the two spaces are plotted. A set of two points has been connected with a line. **C** Relationships between differences in pleasantness scores and eating time. Differences were obtained by subtracting the data under the UCS from those under the CS. Data from all participants have been plotted together. The black line shows a linear fit. The asterisk denotes that differences in pleasantness scores correlate with differences in eating time. *n* = 13, *R*^*2*^ = 0.615, *F* = 20.2, *P* = 9.1 × 10^− 4^. Note that all the points are located in the fourth quadrant

Subjective feelings of pleasantness were compared between the cases of the “virtual cafe space” and the “virtual forest space.” All scores rated by the individual participants in the two virtual spaces were plotted, as shown in Fig. 2A. For all participants, pleasantness scores for “virtual cafe space” were higher than those for “virtual forest space”. The differences in the pleasantness scores between the two cases were significant [Origin8; OriginLab Co., USA; *n* = 13; “virtual cafe space”, 2.0 ± 0.84 (mean ± SD); “virtual forest space”, − 1.6 ± 0.81; paired t-test, *P* = 1.9 × 10^− 6^; Fig. 2A].

Total eating times (sec) were compared between the cases of “virtual cafe space” and “virtual forest space”. The eating times measured by individual participants in the two virtual spaces were plotted as depicted in Fig. 2B. For all participants, the eating time for the “virtual forest space” was longer than that for the “virtual cafe space”. The differences in eating times between the two cases were significant (Origin8; OriginLab Co., USA; *n *= 13; “virtual cafe space”, 99.3 ± 13.7; “virtual forest space”, 107.2 ± 14.3; paired t-test, *P* = 6.0 × 10^− 5^; Fig. 2B).

All participants rated the “virtual cafe space” as positive and the “virtual forest space” as negative. Thus, although there were individual differences in the magnitude of emotion, the data confirmed that all the participants felt that the “virtual cafe space” was comfortable, and the “virtual forest space” was uncomfortable. Thereon, we denoted the “virtual cafe space” as the “comfortable space (CS)”, and the “virtual forest space” as the “uncomfortable space (UCS)”.

In the present study, we focused on the relationship between emotions and eating behavior. Pleasantness scores under the UCS condition were subtracted from those in the CS condition, and we defined them as the “difference in pleasantness score”. In the same way, total eating times under the “UCS” were subtracted from those under the “CS”, and we defined them as “differences in eating time”. Figure 1C shows the relationships between the “difference in pleasantness score” and “difference in eating time”. We examined the correlation between them and found a clear correlation (*n *= 13, *R*^*2*^ = 0.615, *F* = 20.2, *P* = 9.1 × 10^− 4^). The data obtained from all participants were located in the fourth quadrant in the graph, showing that all participants felt pleasantness under the “CS”, and all participants took more time to eat in the “UCS”. In addition, the correlation analyses revealed that when more participants felt comfortable under the “CS”, the time taken to eat in the “US” was longer (see Fig. 7). Thus, the feeding behavior may have been affected by emotions.

### Differences in EEG activities under the “CS” and the “UCS”

**Fig. 3:**
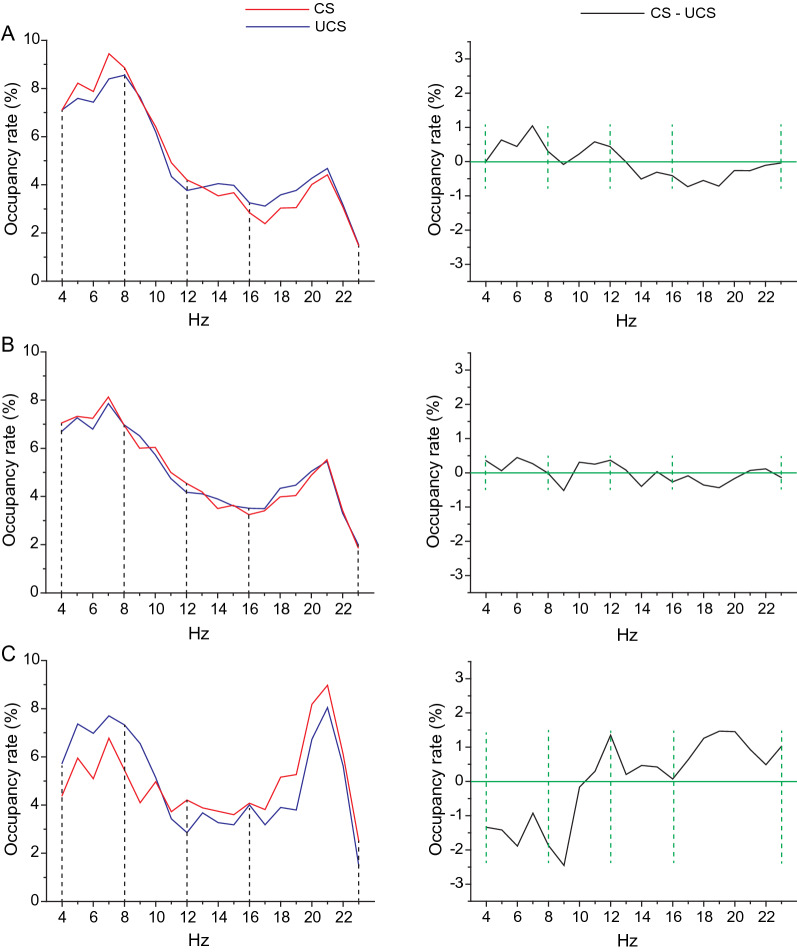
Comparison of the frequency of occupancy rates between the two emotional circumstances. **A**–**C** Comparison of electroencephalogram (EEG) frequency activities during eating in the two virtual spaces. Three representative patterns of occupancy rate profiles under the CS (red line) and UCS (black line). The profiles were obtained by Fast Fourier Transform (FFT) analysis of the EEG signals (see Methods). Left: The two profiles are superimposed, and the four frequency bands that we focused on in the present study are shown. The four frequency bands are as follows: 4–8 Hz (theta), 8–12 Hz (alpha), 12–16 Hz (low beta), and 16–23 Hz (beta). Right: The profile under the UCS was subtracted from that under the CS to investigate the differences in EEG frequency activities. Note that areas surrounded by the line of profile and green line were calculated for every frequency band, and the differences in EEG frequency activities under the CS and UCS were named “differences in areal occupancy rate,” and the “differences in areal occupancy rate,” and used for analyses

EEG signals were recorded from the same 13 participants, and frequency analyses were performed. Profiles of the occupancy rate of EEG frequencies under the “CS” and those under the “UCS” were obtained from all participants. The inter-individual differences of the profiles were confirmed as follows: the representative profiles of “occupancy rates” under the two virtual spaces were superimposed, as shown in Fig. 3A-left, 3B-left, and 3C-left. In the case of Fig. 3A-left, the profile in the “CS” was different from that in the “UCS”. In the case of Fig. 3C-left, the profiles in the two virtual spaces were also different, but the emergence pattern of the EEG was different from the case of Fig. 3A-left. In another case, almost the same patterns of profiles in the two virtual spaces were observed (Fig. 3B-left). Thus, the patterns of EEG emergence in the two virtual spaces varied across individual participants.

To assess brain activity in the four frequency bands, theta, alpha, low-beta, and beta bands, we calculated the areas surrounded by the lines of occupancy rates, the *x*-axis, and the dotted lines that show the frequency classification, and termed the result, the “areal occupancy rate” (Hz × %). The “areal occupancy rate” was calculated for individual participants in the two virtual spaces. In the present study, we estimated differences in EEG activities between the two virtual spaces as “differences in areal occupancy rate” between the two virtual spaces in the respective frequency bands. Figure 3A-right, 3B-right, and 3C-right shows profiles obtained by subtraction of the profile under the “UCS” from that under the “CS”. The areas surrounded by the subtracted lines, the X-axis (green lines), and the dotted lines are equivalent to “differences in areal occupancy rate”. Hereafter, the values of these areas were used for analyses.

### Adaptation of EEG activities in the relationship between emotions and feeding behaviors

**Fig. 4:**
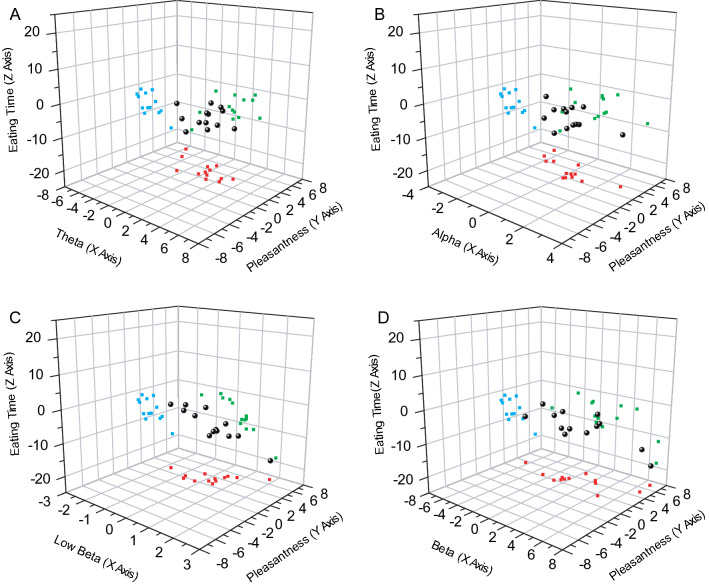
Relationships between the three items of pleasantness scores, frequency occupancy rates, and eating times. Individual values of “differences in pleasantness score,” “differences in eating time,” and “differences in areal occupancy rate” are plotted together as three dimensional graphs. Four graphs are arranged according to the respective frequency band. **A** Theta band, **B** Alpha band, **C** Low Beta band, **D** Beta band. The X-axis represents “differences in areal occupancy rate,” the Y-axis represents “differences in pleasantness score,” and the Z-axis represents “differences in eating time.” The ball-like small black circles indicate the individual values. Relationships between “differences in areal occupancy rate” and “differences in pleasantness score” are projected on the X–Y plane, as small red squares. The relationships between “differences in areal occupancy rate” and “differences in eating time” are projected on the X–Z plane as small green squares. Relationships between “differences in pleasantness score” and “differences in eating time” are projected on the Y–Z plane, as small blue squares. Note the interrelated relationships between the three different comparisons plotted on the three-dimensional graph

The goal of the present study was to elucidate how circumstances affect feeding behavior by altering brain activity and emotions. Analyses of the aforementioned pleasantness scores and eating times (Fig. 2C) suggested a correlation between emotions and feeding behaviors. We then tried to adapt the EEG activities to the relationships, between the emotions and feeding behaviors. Values of differences in areal occupancy rate, differences in pleasantness score, and differences in eating time were plotted in 3-dimensional graphs for every frequency band (Fig. 4A–D).

In these 3-dimensional graphs, the small blue square points in the Y–Z plane represent relationships between the differences in pleasantness score and differences in eating time, and the graph of the Y–Z plane is the same as the 2-dimensional graph in Fig. 2C. Similarly, the small red square points in the X–Y plane represent relationships between the differences in areal occupancy rate and differences in pleasantness score. The small green square points in the X–Z plane represent the relationships between the differences in areal occupancy rate and differences in eating time. These three-dimensional graphs make it easy to depict the relationships between the three different parameters.

Considering this comprehensively, the scattered points seem to be arranged in a relatively linear manner in all the three planes, X–Y, Y–Z, and Z–X in Fig. 4C. The data suggested that brain activity at low-beta frequencies may correlate with both emotions and feeding behaviors (Fig. 4C). Similarly, in Fig. 4A, the scattered points also seem to be arranged in a relatively linear manner in each of the three planes. The brain activity in the theta frequency seems to correlate with both emotions and feeding behaviors (Fig. 4A). Thus, we thereon focused on both the theta and the low-beta frequencies. However, the precise relationships remain unclear at this stage.

### Correlation between EEG activities and pleasantness scores

**Fig. 5:**
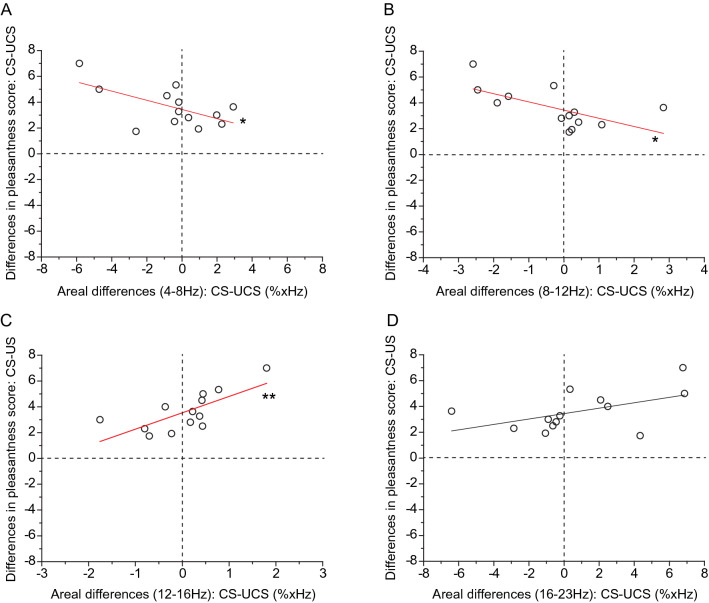
Relationships between individual differences in pleasantness score and areal differences of occupancy rate in the respective frequency band. **A** 4–8 Hz, **B** 8–12 Hz, **C** 12–16 Hz and **D** 16–23 Hz. Each data point represents the differences in the pleasantness score and the areal differences in the occupancy rate. These two-dimensional graphs are the same as those of the X–Y planes, as shown in Fig. 4. Data from all participants are plotted together. The black line shows a linear fit. The red regression line with asterisks denotes that the areal differences in occupancy rates correlate with differences in pleasantness scores. *n* = 13, A: *R*^*2*^ = 0.305, *F* = 6.267, *P* = 0.029; B: *R*^*2*^ = 0.344, *F* = 7.29, *P* = 0.021; C: *R*^*2*^ = 0.474, *F* = 11.82, *P* = 0.006; D: *R*^*2*^ = 0.196, *F* = 3.92, *P* = 0.073

To clarify whether EEG activities relate to feelings of pleasantness in the present experimental protocols, we investigated the correlation between the “differences in areal occupancy rate” and “differences in pleasantness score.” The data obtained from all the participants were plotted, as shown in Fig. 5. In cases of all the frequency bands, the data points are located in the first and second quadrants in the graph, showing that, regardless of the “differences in areal occupancy rate,” the pleasantness score in the “CS” was more than that in the “UCS.” Thereon, the correlation between the “differences in areal occupancy rate” and “differences in pleasantness score” was examined in each respective frequency band. The results were as follows, in the case of theta frequency band: *n* = 13, *R*^*2*^ = 0.305, *F* = 6.27, *P* = 0.029 (Fig. 5A); alpha frequency band: *n* = 13, *R*^*2*^ = 0.344, *F *= 7.29, *P* = 0.034 (Fig. 5B); low-beta frequency band: *n* = 13, *R*^*2*^ = 0.474, *F* = 11.82, *P* = 0.005 (Fig. 5C), and beta frequency band: *n* = 13, *R*^*2*^ = 0.196, *F *= 3.92, *P* = 0.07 (Fig. 5D).

These analyses suggest that the negative weak correlation between the “differences in areal occupancy rate” and the “differences in pleasantness score” for the theta and alpha frequency band shows that the magnitude of the theta and alpha band brain activities is likely to affect the magnitude of pleasantness feeling. Indeed, with the more prominent theta and alpha activities in the “UCS” than in the “CS,” the pleasantness feeling was markedly higher in the “CS” than that in the “UCS”. Whereas, with the more prominent theta and alpha activities in the “CS” compared with that in the “UCS,” the gaps in the pleasantness feeling between the two virtual spaces were small (Figs. 5A, B, 7).

As for the low-beta frequency band, there was a positive clear correlation between “differences in areal occupancy rate” and “differences in pleasantness score,” which shows that the magnitude of the low-beta frequency band brain activity may affect the magnitude of pleasantness feeling. The pleasantness feeling in the “CS” was markedly higher than that in the “UCS” with the more prominent low-beta activity in the “CS” compared with that in the “UCS”. However, the gaps in the pleasantness feeling between the two virtual spaces were small with the more prominent low-beta activity in the “UCS”, than in the “CS” (Figs. 5C, 7).

### Correlation between EEG activities and feeding behaviors

**Fig. 6:**
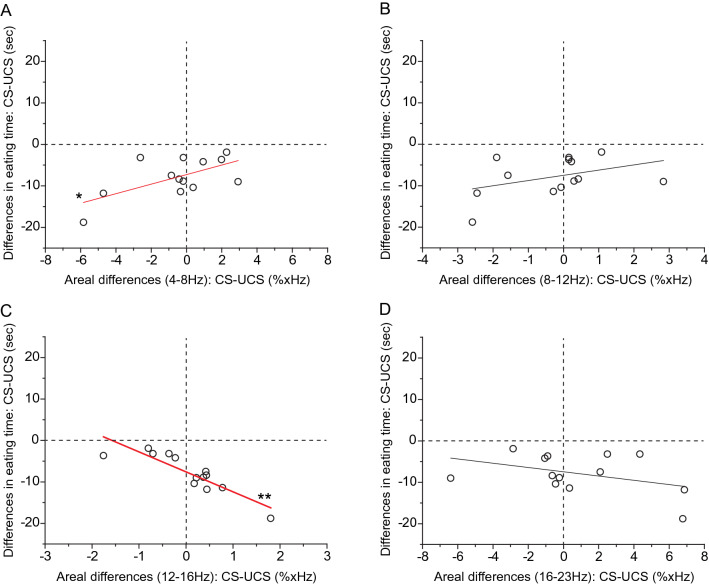
Relationships between individual differences in eating time and areal differences of occupancy rate in the respective frequency band. **A** 4–8 Hz, **B** 8–12 Hz, **C** 12–16 Hz and **D** 16–23 Hz. Each data point represents the differences in eating time and the areal differences in the occupancy rate. These two-dimensional graphs are the same as those of the X–Z planes, as shown in Fig. 4. Data from all participants are plotted together. The black line shows a linear fit. The red regression line with asterisks denotes that the areal differences in occupancy rates correlate with differences in eating time. *n* = 13, **A** *R2* = 0.341, *F* = 7.21, *P* = 0.021; **B** *R*^*2*^ = 0.086, *F* = 2.14, *P* = 0.171; **C** *R*^*2*^ = 0.762, *F* = 39.45, *P* = 5.99 × 10^− 5^; **D** *R*^*2*^ = 0.088, *F *= 2.157, *P* = 0.17

To clarify whether EEG activities related to eating behavior in the present experimental protocol, we investigated the correlation between “differences in areal occupancy rate” and “differences in eating time.” The data obtained from all the participants were plotted, as shown in Fig. 6. In all cases of frequency bands, the data points locate the third and fourth quadrants in the graph, showing that, regardless of the “differences in areal occupancy rate,” the eating time in the “CS” was less than that in the “UCS”. Then, the correlation between “differences in areal occupancy rate” and “differences in eating time” was examined. The results were as follows, in the case of theta frequency band: *n* = 13, *R*^*2*^ = 0.341, *F* = 7.21, *P* = 0.021 (Fig. 6A); alpha frequency band: *n* = 13, *R*^2^ = 0.086, *F* = 2.14, *P* = 0.17 (Fig. 6B); low-beta frequency band: *n* = 13, *R*^*2*^ = 0.76, *F* = 39.45, *P* = 5.99 × 10^–5^ (Fig. 6C); beta frequency band: *n* = 13, *R*^*2*^ = 0.088, *F* = 2.16, *P* = 0.17 (Fig. 6D).

**Fig. 7:**
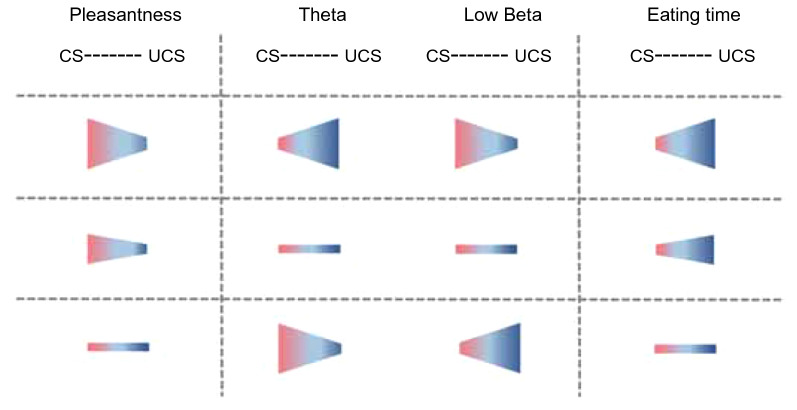
Summary of the relationships between emotion, EEG activity, and feeding behavior. The size of the horizontal image of the trapezoid shows the relative differences between the numerical values acquired in the two circumstances: comfortable and uncomfortable spaces. The relative sizes were based on the correlation analysis. The length of the vertical lines of the trapezoid shows the relative relationship between the two virtual emotional circumstances. In some cases, there were almost no differences between the values in the two circumstances. According to these classifications, the upper column was named, Type 1, the second column was Type 2, and the bottom column was Type 3. *CS* comfortable space, *UCS* uncomfortable space; Light red: comfortable side; Blue: uncomfortable side

These analyses suggest that for the theta frequency band, there was a positive weak correlation between “differences in areal occupancy rate” and “differences in eating time”, which shows that the magnitude of theta band brain activities may affect the total eating time. With more prominent theta activities in the “UCS” compared with that in the “CS”, the eating time in the “UCS” was markedly longer than that in the “CS”. When the theta activities in the “CS” were more prominent compared with that in the “UCS”, the gap in eating time between the two virtual spaces was small (Figs. 6A, 7).

As for the low-beta frequency band, there was a clear negative correlation between “areal differences” and “differences in eating time,” which shows that the magnitude of the low-beta band brain activity may affect the total eating time. With the more prominent low-beta activity in the “CS” compared with that in the “UCS”, the eating time in the “UCS” was markedly prolonged than that in the “CS”. With the more prominent low-beta activity in the “UCS” compared with that in the “CS”, the gap in eating time between the two virtual spaces was small (Figs. 6C, 7). As for other frequency bands, there were no correlations between them.

### Ascertaining prospective classification from the correlation analyses

Based on the results of precise analyses, we noted a tendency among inter-individual differences. Since the EEG activities of theta frequency bands were weakly correlated with pleasantness and eating time, and low-beta frequency bands were correlated with pleasantness and eating time, we focused solely on these two frequency bands. We approximately divided several characteristics into three patterns, as follows (Fig. 7): Type 1: When the relative value of pleasantness in the “CS” was markedly higher, the theta activity in the “UCS” was prominent, and simultaneously the low-beta activity in the “CS” was prominent, then, the eating time in the “UCS” was markedly prolonged. Type 2: When the relative value of pleasantness in the “CS” was moderately high, the gap in theta activity between the two virtual spaces was small, the gap in low-beta activity between the two virtual spaces was also small, and the eating time in the “UCS” was comparatively prolonged. Type 3: When the gap of pleasantness between the two virtual spaces was small, the theta activity in the “CS” was prominent, and simultaneously, the low-beta activity in the “UCS” was prominent; then, the gap in eating time between the two virtual spaces was small. Thus, individual variations of relationships between pleasantness, EEG activity, and eating behaviors can possibly be classified into three such patterns.

## Discussion

### Outlines of the results in the present study

The outline of our findings is as follows. First, we established a clear correlation between the feeling of pleasantness and eating time, which shows that the relationship between the two items is simple. Second, focusing on theta and low-beta frequency bands, both EEG frequency band activities respectively correlated with the magnitude of pleasantness and eating time. Comprehensively considering these results, we classified these several combinations of correlations into approximately three patterns of characteristics, as shown in Fig. 7. The reason that grouping might not be simple could be that, even under the same virtual space, EEG frequency profiles that emerged in the sessions were markedly different across the participants. However, the grouping should be easy to understand by considering linear fittings in the graphs of figures, where regression lines are drawn in two-dimensional graphs. In the case of Fig. 2C, the regression lines with data points located within the fourth quadrant, and the point where the regression lines cross the Y-axis (Y-intercept) were almost zero. Therefore, the relationship between pleasantness and eating behavior may be simply understood. In contrast, in the cases of Figs. 5 and 6, the regression lines with data points are located across the first and second quadrants (Fig. 5A, C), or across the third and fourth quadrants (Fig. 6A, C), and the point of the Y-intercept is far from zero in the graphs. Together with these observations, a plausible interpretation is as follows: When the range of emotion is wider between the comfortable and uncomfortable circumstances, the theta frequency activities are prominent in uncomfortable circumstances, whereas the low-beta frequency activities are prominent in comfortable circumstances, as it takes more time to eat in uncomfortable circumstances (Type 1, see Fig. 7). In contrast, when the range of emotion is small between the two circumstances, theta frequency activities are prominent in comfortable circumstances, while low-beta frequency activities are prominent in uncomfortable circumstances, and then the eating behavior is more resistant in being influenced by the emotional circumstances (Type 3, Fig. 7). When the range of emotion was moderate between the two circumstances, prolongation of the eating time under uncomfortable circumstances was moderate, and in that case, there was almost no difference in brain activity between the two circumstances in both the theta and low-beta frequency bands (Type 2, Fig. 7). Considering comprehensively, these results suggest possible relatedness between emotion, feeding behavior, and brain activity. The classification in Fig. 7 shows how circumstances affect feeding behavior through the EEG frequency activity.

### Generation of emotion

In the present study, we prepared two different virtual spaces using photographs and sound. One was the virtual space with chocolate in a cafe room and consonant music (“virtual cafe-space”), and the other was a virtual space with photos of a forest with many bugs and sounds of flying insects (“virtual forest-space”). In the “virtual cafe-space,” all participants rated the pleasantness scores as positive, whereas all participants rated pleasantness scores as negative in the “virtual forest -space.” In addition, all participants rated the pleasantness scores for the “virtual cafe-space” higher than those for the “virtual forest-space.” Thus, in subjective emotional aspects, all patients felt comfortable in the “virtual cafe-space,” and uncomfortable in the “virtual forest-space” in the same way. However, in the objective measures of EEG, markedly different patterns were observed across the participants.

In the present study, the participants received visual, auditory, gustatory, olfactory, and oral somatosensory information. Among the sensory information, visual and auditory information may largely affect the generation of positive or negative feelings in the present experimental protocols. The mechanism of emotion generation in the brain is complex. The output of mental feelings may be mainly generated by the region between the prefrontal and limbic systems based on not only instinctive emotions but also subjective experiences [7, 14, 20]. Before making the final output of emotional feeling, sensory information processing by neocortical regions participates in generating emotions within the limbic systems [7, 15]. Previous studies have reported that when listening to music, distributed brain areas perform music information processing, such as the amygdala, hippocampus, right central striatum, auditory cortex, pre-supplementary motor area, cingulate cortex, and orbitofrontal cortex [21, 22]. During music listening, the frontal lobe modulates valenced experiences, and the parieto-temporal region contributes to emotions [23]. As for unpleasant music, the hippocampus, para-hippocampal gyrus, temporal poles, and amygdala get activated [24]. With regard to visual-evoked emotions, pleasant pictures activate the right caudate head, extending to the nucleus accumbens and the left dorsolateral cortex. However, unpleasant pictures activate the right amygdala and left caudate body [25]. In particular, variations in amygdala reactivity induce inter-individual variations in fear learning [26]. Furthermore, it has been reported that neural representations of diverse emotional experiences during video viewing are high-dimensional, categorical, and distributed across transmodal brain regions, centered in the default mode network [27]. Thus, visual and auditory information generate emotions through various pathways of sensory information processing based on instinctive responses and subjective experiences.

### Emotion and the EEGs

Thus, before a final feeling is reached, many cortical and subcortical areas are activated. Recent studies have reported relationships between mental conditions and EEG frequency bands [28, 29]. In addition, it has been demonstrated that dynamic causal connectivity exists in the brain network [30], and that frequency bands can be classified based on functional connectivity [31]. Different patterns of functional connectivity between brain sites are associated with different emotional states and EEG frequency bands. Therefore, if the processes or strategies of producing emotions differed between our participants, the different inter-individual EEG patterns could be observed. Indeed, in the present study, patterns of EEG emergence varied widely among participants in the emotional aspects.

Recently, the frontal midline (FM)-theta rhythms were reported to reflect mental concentration as well as meditative states [14, 17, 32]. In addition, FM-theta appears in the frontal lobe when image-related emotions are produced [33]. In the present experimental protocols, the theta rhythm might be dominantly related to the generation of emotions. When theta frequency activity is prominent under uncomfortable circumstances, the range of emotions widens (Type 1). In contrast, when theta frequency activity is prominent under comfortable circumstances, the range of emotion becomes smaller (Type 3). These differences may depict an inter-individual difference that depends on how theta frequency emerges between comfortable and uncomfortable virtual spaces. Indeed, in the case of Type 1 participants, emotion may be easily moved in emotional virtual spaces, whereas in the case of Type 3 participants, it may not be easily moved. Thus, theta frequency may be related to inter-individual differences in emotion modulation.

### Feeding behavior, emotion, and the EEG

Feeding behavior is affected by the internal state of the body, such as hunger, taste, smell, mood, and environment [34]. Under these conditions, feeding behavior is mainly controlled by the feeding center in the lateral hypothalamus. The lateral hypothalamus receives neural inputs from various areas such as the amygdala, pallidus, prefrontal cortex, and arcuate nucleus [7, 11]. The lateral hypothalamus sends information to the mastication central pattern generator in the brainstem. Thus, feeding behaviors may be controlled by the lateral hypothalamus, where various types of information converge. Recently, the central nucleus of the amygdala (CeA) was reported to directly or indirectly control feeding behavior. The CeA also receives neural inputs from food-intake-related areas in the brain, related to appetite, food palatability, and taste, among others, to modulate feeding behaviors [9]. Indeed, the CeA sends efferent projections to the trigeminal nerve, lateral hypothalamus, parabrachial nuclei, and ventral tegmental area [7]. Thus, feeding behavior is controlled by various roots.

In the present experimental protocol, especially in the “uncomfortable virtual spaces,” the amygdala and prefrontal cortex would have strongly modulated the lateral hypothalamus. Indeed, all participants rated the “uncomfortable virtual spaces” negatively, and with a result that eating times in uncomfortable spaces were prolonged compared with under the comfortable space (Fig. 2C). Thus, all participants exhibited the same features in their feeding behaviors. However, different strategies for producing EEG must be used across participants, and EEG patterns have emerged differently during eating.

From the correlation analyses, among the inter-individual varieties of EEG frequency patterns, distinctive properties across individuals were found in theta and low-beta frequency bands (see Figs. 5, 6, 7). The relative eating times between the two virtual spaces may depend on how these two frequencies emerge in the emotional spaces. The results suggest that, across the subjects, there is an individual brain function related to producing theta and low-beta waves, and the individual function may be concerned with producing individual emotions and modulating feeding behaviors.

Recently, it was reported that beta oscillations are closely related to motor movement [35]. In particular, after the intended movement, beta oscillations markedly emerged. In this situation, cortical beta activities are associated with sensorimotor processing [36–40]. In the present experimental protocols, the low-beta frequency, which is a low-frequency zone of beta frequency, might be dominantly involved in the modulation of feeding behavior. When low-beta frequency activity is prominent under comfortable circumstances, it takes a shorter time in the comfortable circumstance (Type 1). On the contrary, when low-beta frequency activity was prominent under uncomfortable circumstances, the gap in the eating time between the two circumstances was small (Type 3). The differences may show an inter-individual difference that depends on how low the beta frequency emerged between the comfortable and uncomfortable virtual spaces. Nevertheless, a low-beta frequency may contribute to the modulation of eating behaviors. The eating time of participants of Type 1 may easily be prolonged in uncomfortable virtual spaces compared to those of Type 3. However, in participants with Type 3, the eating times may not be easy to modulate in either virtual space.

Figure 7 shows the entire relationships between the mental condition, brain activities based on EEG frequency, and feeding behaviors under comfortable and uncomfortable circumstances. These relationships predict the flows of “cause-and-effect” of eating behavior under the emotional spaces. Considering the previous studies mentioned above, EEG frequency activities of the theta band may cause a greater magnitude of emotions, and at the same time, the activities of the low-beta frequency band may cause changes in eating patterns. Thus, theta and low-beta frequencies may play an important role in modulating the feeding center in the brain and the feeding behaviors. The present study did not directly demonstrate the roles of the theta and low-beta frequency bands. We used the methods of comparing emergence ratios of the EEG frequency band between emotional circumstances, which enabled us to compare the participants. One of the interesting findings in the present study is that focusing on the emergence ratio of theta and low-beta frequency bands, there is an inverse relationship between them in individual participants. Therefore, this finding may affirm that different areas in the brain may be concerned with creating emotions and modulating feeding behavior between participants, which may produce individuality.

### Autonomic nervous systems and environments during feeding

In the present study, participants performed the tasks under emotional circumstances. Therefore, we should consider the involvement of the autonomic nervous system (ANS), as ANS activity is strongly related to emotions [41–43]. Activation of the ANS influences various biosignals, such as blood pressure, breathing rate, heart rate, EEG frequency, and salivary amylase [42, 44–46].

Focusing on saliva, changes in emotions affect not only saliva flow but also the components of the organic substances of saliva [47, 48]. Therefore, several salivary components are used as markers for the activation balance of ANS and mental conditions. In the present study, however, we did not measure salivary components. However, all participants commented that the flow of saliva decreased in uncomfortable environments and increased in comfortable environments. In general, when the parasympathetic nervous systems dominate, the flow of saliva increases [49], suggesting that environments during “Session 1” may cause increasing parasympathetic activation. When chocolate is sucked on, in a comfortable environment, an increase in salivary flow may facilitate chocolate melting in the mouth, making it easier to eat chocolate. However, in our study, eating times tended to increase in comfortable environments. In general, an increase in saliva flow increases taste sensation, flavor perception, and saliva-food interactions. [50, 51]. These factors may contribute to an increase in feeding pleasure, which may prolong the eating time. In terms of nutrition, comfortable environments may be crucial for digestion and absorption by increasing parasympathetic activation.

## Conclusions

Feeding behavior may be easily affected by mental conditions. Examining relationships between mental conditions, feeding behaviors, and brain activities in comfortable and uncomfortable virtual spaces, we found that the theta, alpha, and low-beta band activities may guide the strength of mental conditions, whereas manners of theta and low-beta band activities may guide feeding behaviors. Thus, theta and low-beta bands may be crucial and relevant waves for feeding behaviors under emotional circumstances that are influenced by alterations in mental conditions.

## Data Availability

Data could be obtained upon request to the corresponding author.

## Abbreviations

EEG: Electroencephalogram
CeA: Central nucleus of the amygdala
PFC: Prefrontal cortex
